# Maternal regulation of chromosomal imprinting in animals

**DOI:** 10.1007/s00412-018-00690-5

**Published:** 2019-02-05

**Authors:** Prim B. Singh, Victor V. Shloma, Stepan N. Belyakin

**Affiliations:** 1 Nazarbayev University School of Medicine, 5/1 Kerei, Zhanibek Khandar Street, Astana, Z05K4F4 Kazakhstan; 2 Epigenetics Laboratory, Department of Natural Sciences, Novosibirsk State University, Pirogov str. 2, Novosibirsk, 630090 Russian Federation; 3 Genomics Laboratory, Institute of Molecular and Cellular Biology SD RAS, Lavrentyev ave, 8/2, Novosibirsk, 630090 Russian Federation

**Keywords:** Parent-of-origin effects, Chromosomal imprinting, Genomic imprinting, Heterochromatin, Epigenetics, Non-coding RNA, Germ-line differentially methylated regions, H3K9me3:HP1:H4K20me3 pathway, *Mus musculus*, *Sciara coprophila*, *Plannococcus citri*

## Abstract

Chromosomal imprinting requires an epigenetic system that “imprints” one of the two parental chromosomes such that it results in a heritable (cell-to-cell) change in behavior of the “imprinted” chromosome. Imprinting takes place when the parental genomes are separate, which occurs during gamete formation in the respective germ-lines and post-fertilization during the period when the parental pro-nuclei lie separately within the ooplasm of the zygote. In the mouse, chromosomal imprinting is regulated by germ-line specific DNA methylation. But the methylation machinery in the respective germ-lines does not discriminate between imprinted and non-imprinted regions. As a consequence, the mouse oocyte nucleus contains over a thousand oocyte-specific germ-line differentially methylated regions (gDMRs). Upon fertilization, the sperm provides a few hundred sperm-specific gDMRs of its own. Combined, there are around 1600 imprinted and non-imprinted gDMRs in the pro-nuclei of the newly fertilized zygote. It is a remarkable fact that beginning in the maternal ooplasm, there are mechanisms that manage to preserve DNA methylation at ~ 26 known imprinted gDMRs in the face of the ongoing genome-wide DNA de-methylation that characterizes pre-implantation development. Specificity is achieved through the binding of KRAB-zinc finger proteins to their cognate recognition sequences within the gDMRs of imprinted genes. This in turn nucleates the assembly of localized heterochromatin-like complexes that preserve methylation at imprinted gDMRs through recruitment of the maintenance methyl transferase Dnmt1. These studies have shown that a germ-line imprint may cause parent-of-origin-specific behavior only if “licensed” by mechanisms that operate post-fertilization. Study of the germ-line and post-fertilization contributions to the imprinting of chromosomes in classical insect systems (*Coccidae* and *Sciaridae*) show that the ooplasm is the likely site where imprinting takes place. By comparing molecular and genetic studies across these three species, we suggest that mechanisms which operate post-fertilization play a key role in chromosomal imprinting phenomena in animals and conserved components of heterochromatin are shared by these mechanisms.

## Maternal regulation of chromosomal imprinting in mice

Pre-genome-wide analyses showed that methylation of cytosines in CpG dinucleotides is crucially involved in chromosomal imprinting in the mouse (Ferguson-Smith 2011). This was first demonstrated in mice deficient for the maintenance DNA methyl transferase Dnmt1 that methylates the un-methylated cytosine of CpG dinucleotides of newly replicated DNA (Bestor et al. 2015). Mutation of Dnmt1 resulted in dys-regulation of imprinted genes (Li et al. 1993). The loss of imprinting was associated with the loss of CpG methylation at germ-line differentially methylated regions (gDMRs) that are associated with imprinted genes. Imprinted gDMRs are methylated either in the female (maternally-imprinted) or male (paternally-imprinted) germ-lines. Maternally imprinted gDMRs have a CpG density and GC richness that categorizes them as members of CpG-rich genomic regions known as CpG islands (CGIs), while paternally imprinted gDMRs fall just short of these criteria, but are not atypical (Kobayashi et al. 2006; Tomizawa et al. 2011). To date, there are around 26 (23 maternal and 3 paternal) definitive imprinted gDMRs (Hanna and Kelsey 2014; Wang et al. 2014). Of these ~ 26 imprinted gDMRs, seven have been shown to be imprinting control regions (ICRs) that cause parent-of-origin-specific expression in functional assays (Barlow and Bartolomei 2014; Ferguson-Smith 2011); to avoid the use of too many acronyms, we use the term imprinted gDMRs instead of ICR for the rest of this discussion.

**Fig. 1:**
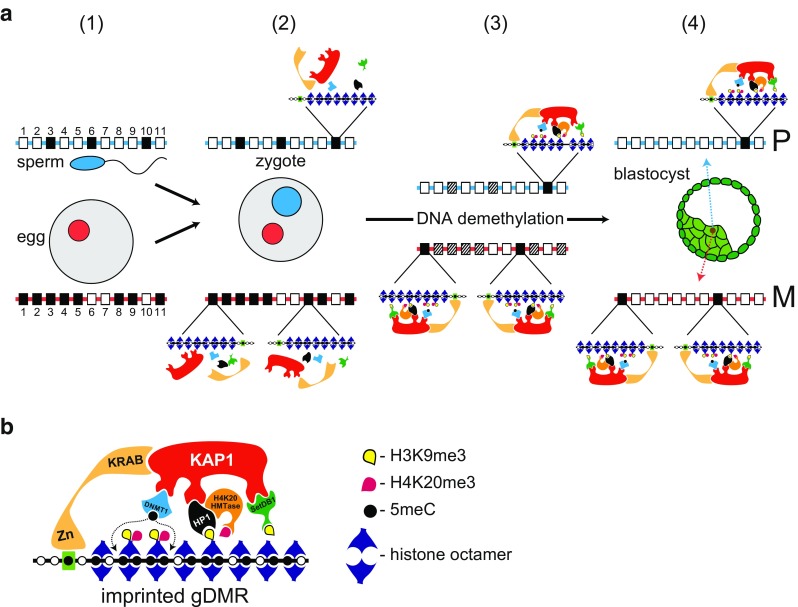
Maternal regulation of chromosomal imprinting in mice. **a** Preservation of methylation at imprinted gDMRs. (1) The paternal (sperm nucleus in blue) and maternal (oocyte nucleus in red) nuclei contain homologous chromosomes that carry CpG islands (CGIs) depicted as rectangles numbered 1 through to 11 on blue (paternal homolog) and red (maternal homolog) lines. Open rectangles represent non-methylated CGIs. Some methylated CGIs are shared (e.g., closed rectangle at position 3 on both parental homologs) and are not gDMRs. Some methylated CGIs are non-imprinted gDMRs (e.g., closed rectangles at position 6 on paternal chromosome and positions 2, 4, 5, 9, and 11 on the maternal chromosome) that will lose their methylation during the DNA demethylation that takes place as embryos pass through preimplantation development. A few methylated CGIs are imprinted gDMRs (closed rectangles at position 10 on the paternal chromosome and 1 and 8 on the maternal homolog) that will retain their methylation status through DNA demethylation. In actuality, there are over a thousand non-imprinted and imprinted gDMRs present in the oocyte nucleus and a few hundred will enter with the sperm (Kobayashi et al. 2012). This difference in number is reflected in the difference in closed rectangles on the maternal (red line) and paternal (blue line) homologs. (2) The maternal (in red) and paternal (in blue) pro-nuclei contain the homologous chromosomes (red and blue lines, respectively) described in (1). Of the ~ 1600 non-imprinted and imprinted gDMRs in the zygote, only a small percentage—the imprinted gDMRs—will preserve DNA methylation in the face of the DNA demethylation that takes place during pre-implantation development (Messerschmidt et al. 2014). The initial assembly of the heterochromatin-like complexes that preserve methylation at imprinted gDMRs takes place in the newly fertilized zygote (see text for details). (3) Preservation of methylation at imprinted gDMRs on the paternal (position 10) and maternal (positions 1 and 8) homologs is due to localized heterochromatin-like complexes at imprinted gDMRs. The complexes preserve DNA methylation at imprinted gDMRs throughout pre-implantation development; non-imprinted gDMRs and methylated CGIs become de-methylated (stippled rectangles). (4) Global levels of DNA methylation reach their lowest point in embryonic nuclei of the blastocyst. However, methylation at imprinted gDMRs is preserved by the heterochromatin-like complexes shown in (3), on the paternal (position 10) and maternal (position 1 and 8) homologs. P denotes paternal homolog and M the maternal homolog. **b** Assembly of localized heterochromatin-like complex at imprinted gDMRs. Methylation of cytosines in CpG dinucleotides (black circles) is preserved by the assembly of a heterochromatin-like complex at imprinted gDMRs. The complex is targeted by the KRAB zinc-finger protein Zfp57 that binds the hexamer motif TGCCGC when the cytosine in the CpG is methylated (black circle in green rectangle). This in turn recruits KAP1, which is a modular protein that acts as a focal point for the recruitment of Setdb1 histone methyltransferase, HP1, and Dnmt1. HP1 binds the H3K9me3 generated by Setdb1 and recruits a H4K20me3 histone methyl-transferase that generates H4K20me3 thus forming the H3K9me3:HP1:H4K20me3 pathway. DNA methylation at the imprinted gDMR is maintained (dotted lines) by Dnmt1. For a full description of the molecular constituents of the heterochromatin-like complex, see Singh (2016)

The discovery that imprinted genes contain gDMRs led to the widely held notion that specific mechanism(s) exist in the germ-line to target imprinted genes. This view was revised with the advent of genome-wide studies because comparison of the sperm and oocyte methylomes revealed many non-imprinted regions that are differentially methylated between the genomes, far above the number of gDMRs associated with imprinted genes. Non-imprinted gDMRs were found to be methylated in much the same way as imprinted gDMRs (Hanna and Kelsey 2014; Stewart et al. 2016). Although the numbers vary between studies, it was found that there were over a thousand imprinted and non-imprinted gDMRs in the oocyte nucleus while a few hundred were present in the sperm, giving a combined total of around 1600 imprinted and non-imprinted gDMRs in the zygote (Kobayashi et al. 2012; Smallwood et al. 2011). The key difference between imprinted gDMRs and non-imprinted gDMRs is that the former are protected from the dramatic DNA demethylation that characterizes pre-implantation development in the mouse (Leseva et al. 2016) (Fig. 1a). It is the mechanism(s) that preserves methylation at imprinted gDMRs in the zygote and preimplantation embryo, at a time when much of the remainder of the genome is being demethylated, which lies at the heart of the imprinting process in mice.

There are non-specific and specific mechanisms that protect imprinted gDMRs from DNA demethylation (Messerschmidt et al. 2014). The non-specific mechanism requires STELLA a protein that does not bind DNA and has specificity for H3K9me2. It has a global role in the protection of the maternal pro-nucleus—which is enriched in H3K9me2 compared to the paternal pro-nucleus—from “active” demethylation (Nakamura et al. 2007, 2012). In the absence of maternal STELLA, loss of 5-methyl-cytosine (5meC) is observed in both pro-nuclei (Wossidlo et al. 2011). Loss of global protection of the maternal pro-nucleus also leads to DNA hypomethylation of the maternally imprinted *Peg1*, *Peg3*, and *Peg10* gDMRs (Nakamura et al. 2007). Two of the three paternally imprinted gDMRs, those associated with *H19* and *Rasgrf1*, retain H3K9me2-marked chromatin during spermatogenesis and protamine exchange and, as a consequence, STELLA binds H3K9me2 and protects both gDMRs against demethylation (Nakamura et al. 2012).

Sequence-specific preservation of methylation at imprinted gDMRs requires the activity of maternally supplied KRAB-zinc-finger proteins (KRAB-Zfps) of which the best described is Zfp57. Loss of maternal-zygotic Zfp57 results in demethylation of many imprinted gDMRs (Li et al. 2008; Quenneville et al. 2011; Zuo et al. 2012); the KRAB-Zfp, Zfp455, has been shown to act in concert with Zfp57 to preserve methylation at imprinted gDMRs (Takahashi et al. 2019). Binding of Zfp57 to imprinted gDMRs is via a hexamer motif TGCCGC where the central CpG dinucleotide is methylated (Anvar et al. 2016; Liu et al. 2012; Quenneville et al. 2011). As shown in Fig. 1a, b, Zfp57 binding nucleates the assembly of a heterochromatin*-*like complex. The initial assembly is likely to take place soon after fertilization using products laid down maternally. The Zfp57 co-repressor KAP1 is found in oocytes and maternal loss affects methylation at gDMRs (Messerschmidt et al. 2012). Likewise, Dnmt1 is found in the ooplasm (Cirio et al. 2008; Kurihara et al. 2008) and loss of maternal-zygotic Dnmt1 leads to loss of methylation at imprinted gDMRs (Hirasawa et al. 2008). The Setdb1 K9-HMTase is found in the pro-nuclei of the zygote (Cho et al. 2012) and maternal loss has a debilitating effect on early development (Kim et al. 2016). HP1 proteins are also found in the oocyte cytoplasm and pro-nuclei of the early embryo (Arney et al. 2002; Probst et al. 2007). Once assembled, the complex preserves methylation at imprinted gDMRs throughout the demethylation phase (Fig. 1a). Later, in embryonic stem (ES) cells derived from the blastocyst, genome-wide studies have shown that Zfp57 binding sites overlap with KAP1, Setdb1, and with *tri*-methylated lysine 9 of histone H3 (H3K9me3) and *tri*-methylated lysine 20 of histone H4 (H4K20me3) peaks (Quenneville et al. 2011; Strogantsev et al. 2015). The presence of H4K20me3 indicates that a H4K20HMTase is recruited to imprinted gDMRs (Pannetier et al. 2008), most likely through a known interaction of H4K20HMTases with HP1 (Schotta et al. 2004); ChIP analyses have shown that HP1 proteins localize to imprinted gDMRs (Pannetier et al. 2008; Quenneville et al. 2011; Regha et al. 2007). A key interaction is that of KAP1 with Dnmt1 (Quenneville et al. 2011; Zuo et al. 2012), which has the effect of recruiting Dnmt1 to imprinted gDMRs thereby maintaining methylation at these sites (Messerschmidt et al. 2014). Dnmt1 may also be recruited to imprinted gDMRs through a known interaction with HP1 proteins, an interaction that has been shown to increase local DNA methylation levels (Smallwood et al. 2007). Specific mechanisms that concentrate Dnmt1 at imprinted gDMRs are necessary because Dnmt1 is scarce in the early embryo as a consequence of the need to de-methylate the embryonic genome in order for proper development to proceed (Messerschmidt et al. 2014).

What is evident from the mouse work is that the molecular machinery that methylates imprinted gDMRs is unlikely to be different to that which methylates non-imprinted gDMRs or, indeed, any CGI. Nevertheless, it is DNA methylation in the context of a specific DNA sequence that provides an “imprint” for sequence-specific mechanisms that operate post-fertilization to recognize and preserve the methylation at imprinted gDMRs. Thus, chromosomal imprinting in mice involves both a germ-line and a post-fertilization component. Study of the relative contribution of these two components in the insect *Sciara coprophila*, where the term chromosomal imprinting was coined (Crouse 1960a), shows that events taking place post-fertilization are likely to make a greater contribution to the imprinting process.

## Maternal regulation of chromosomal imprinting in *Sciara coprophila*

Chromosomal imprinting in *S. coprophila* is complicated, involving both the elimination of the entire paternal chromosome set in primary spermatocytes and the programmed elimination of paternal X chromosomes (Xps) in the soma and germ-line. For detailed descriptions of the *Sciara* chromosome cycle, see Gerbi (1986), Metz (1938), Singh (2016), and Singh and Belyakin (2018). It is unclear whether the site and timing of the “imprinting” events that regulate each of these parent-of-origin-specific behaviors are the same although it has been argued that imprinting in *Sciara* takes place in the pro-nuclei of the newly fertilized zygote (Chandra and Brown 1975). Here, we revisit the evidence in support of that view, in light of more recent advances, mainly to show there may be molecular similarities between imprinting mechanisms across species, but also to focus attention to where molecular techniques could be applied to elucidate the imprinting phenomena in this extraordinary system.

In *Sciara coprophila* males the entire paternal set of chromosomes is eliminated during meiosis I. Consequently, only the maternal chromosomes enter the sperm and these very same chromosomes will be recognized as paternal after fertilization. Meiosis II is orthodox except for a unique feature in there is non-disjunction of the maternal X chromosome (Xm), which makes its way precociously to the monopole in secondary spermatocytes. The Xm-dyad passes into the sperm that is now double-X (XpXp) leading, after fertilization, to the characteristic 3X (XpXpXm) constitution in the zygotic nucleus. Considering the regular chromosomes and supernumerary germ-line limited or “L” chromosomes, the zygote contains 11–12 chromosomes, where the contribution from the female pro-nucleus is 5 chromosomes (3 autosomes, 1 X′ or X chromosome, and 1 L chromosome) and male pro-nucleus 6–7 chromosomes (3 autosomes, 2 identical X chromosomes, and 1–2 L chromosomes). During the embryonic cleavage divisions an extraordinary pattern of programmed chromosome eliminations takes place in cells destined to become the soma. At the 5th–6th embryonic division, the L chromosomes are eliminated. At the 7th–8th, both paternal X chromosomes (Xps) are eliminated from the male soma, while one Xp is eliminated from the female soma. The somatic constitutions are therefore typical, XmO for male and XmXp for female. The germ-line of both sexes is XmXp because one Xp is eliminated from resting germ cells on the first day of larval life. Elimination of the Xp chromosomes is regulated by a controlling element (CE) on the X-chromosome that resides within the rDNA cluster in heterochromomere II (H2) adjacent to the X centromere (Crouse 1960a, 1979; Crouse et al. 1977). The sequence of the CE is not known but H2 contains an additional 30 kb of non-rDNA sequence that may represent the *cis*-acting CE (cited in Gerbi (2007)). The CE not only regulates the elimination of Xp in the soma and germ-line but also the non-disjunction of the Xm by centromere inactivation in secondary spermatocytes and precocious movement of the resultant Xm-dyad to the monopole.

The mechanism(s) that confer the parent-of-origin-specific behavior of the chromosomes has been the subject of intense cytogenetic investigation over several decades. Crouse and colleagues concluded that there were two imprints, one paternal and the other maternal (Crouse 1960a; Rieffel and Crouse 1966). As for the paternal “imprint”, Crouse was particularly interested in finding the stage when the maternal chromosomes that enter the sperm—thereby becoming paternal chromosomes—were likely to be “imprinted” or “marked” for elimination in primary spermatocytes of the next generation. The approach taken was to compare the condensation cycles of the supernumerary L chromosomes and the regular chromosomes (Crouse et al. 1971). The reasoning behind this was that L chromosomes, unlike the regulars, are not subject to imprinting (Crouse et al. 1971; Rieffel and Crouse 1966). And comparison of the condensation cycles of the two kinds of chromosome could indicate the site and timing of the putative paternal imprint. This analysis led to the suggestion that imprinting takes place in the brief window at the end of anaphase in meiosis II, where the greatest disparity exists, with L chromosomes being highly condensed (heteropyknotic)—and thus likely to be resistant to the imprinting process—at the time when the regulars are diffuse (discussed in Crouse et al. (1971)). While this exacting study had identified a stage where a paternal “imprint” might be placed on the maternal homologs to mark them for elimination in sons, this could not be the whole story. This is because elimination of the Xp chromosomes in the soma is solely under maternal control!

Earlier work had shown that the site and timing of the “imprinting” event that leads to elimination of paternal X chromosomes takes place in the egg. This work can be explained simply because *Sciara coprophila* is monogenic (reviewed by Metz (1938)). A given female gives rise to a family all of which are same sex. Females which produce families that contain daughters are XX′ while XX mothers have sons; the X′ chromosome possesses a long para-centric inversion (Crouse et al. 1977). When a single male inseminates two females, one XX′ and the other XX, the outcomes are very different. This is because X- and X′-borne genes condition the cytoplasm of the fertilized egg to cast aside the appropriate number of Xp chromosomes during the 7th–8th cleavage in embryonic development, and it is this elimination that decides whether the soma shall be XX or XO. These observations indicate that, depending on the genetic constitution of the mother, any putative imprint on the paternal X chromosomes that enters the egg in the sperm may be registered by the maternal cytoplasm leading to elimination, ignored, or even erased. Indeed, Chandra and Brown (1975) have suggested that regulation of imprinting by the egg cytoplasm is sufficient to explain the imprinting phenomena in *Sciara*.

**Fig. 2:**
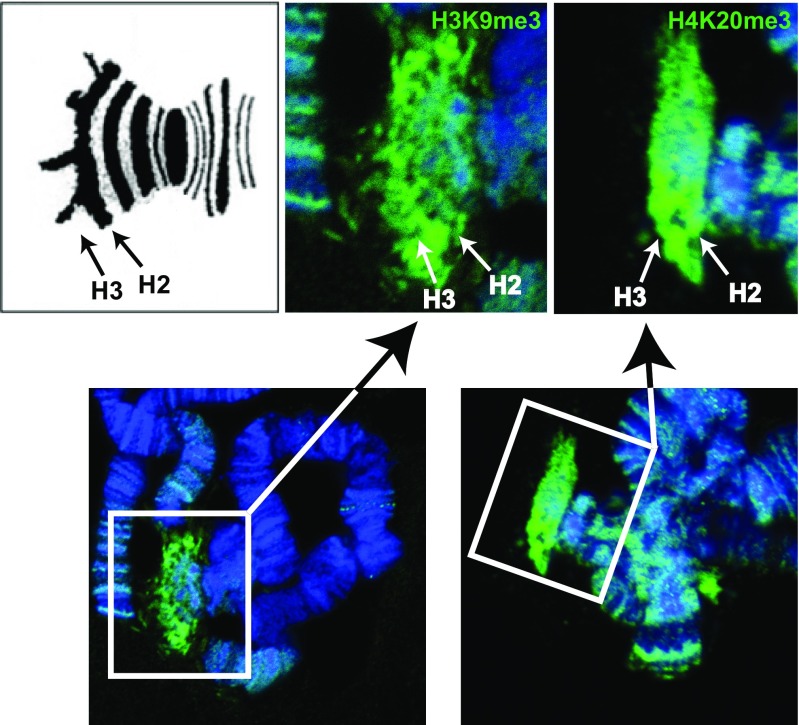
H3K9me3 and H4K20me3 are enriched at the CE on polytene chromosomes. The bottom two panels show partial polytene chromosomes spreads, where rectangles encompass the termini of the X-chromosomes that were labeled with antibodies specific for H3K9me3 (left) and H4K20me3 (right). In the top row, the left panel is a schematic representation of a polytene X chromosome centromeric end (Crouse et al. 1977; Gabrusewycz-Garcia 1964). The arrows show the positions of heterochromomeres H3 and H2; H2 contains the CE. The middle panel is a higher magnification of the distribution of H3K9me3 on the polytene X chromosome end. H3K9me3 is enriched on H3 and H2 heterochromomeres. The right panel is a higher magnification of the distribution of H4K20me3 on the polytene X chromosome end. H4K20me3 is enriched on H3 and H2 heterochromomeres. The staining of *Sciara coprophila* polytene chromоsomes with anti-H3K9me3 and -H4K20me3 antibodies was according to Cowell et al. (2002) and Kourmouli et al. (2004), respectively

Further insight into the nature of the imprint came from studies on the X-autosome translocations that were used to map the CE (Crouse 1960a, b). These translocations increase the frequency of 3:1 disjunction during oogenesis in XX′ and XX mothers and the production of a significant number of eggs that receive two copies or no copies of the X chromosome rather than just one copy (Crouse 1960b). When such eggs are fertilized by wild-type males exceptional offspring are produced, where sons are produced from XX′ mothers that normally have daughters only and daughters from XX mothers that should have sons. These studies have confirmed that regardless of the number of X chromosomes in the egg and therefore the embryo the X′ chromosome ensures that just one paternal X chromosome is eliminated in embryogenesis while from XX mothers, two paternal X chromosomes are eliminated (Crouse 1960b, 1966). Since the exceptional offspring have the somatic chromosome number expected of the sexes, sex of the soma is determined by the chromosomes. It follows that conditioning of the egg cytoplasm does not influence the sex of the embryonic soma per se, but rather just counts the number of paternal Xs that are to be eliminated. Importantly, the timing of the eliminations of the Xps indicates that the “imprint” is *heritable* through several cell generations (Crouse 1960a, b). This cell-to-cell inheritance of the imprint has been posited as having the characteristics of heterochromatin (Singh et al. 1991). This notion is supported by more recent immunocytological studies that have shown that the CE on polytene chromosomes—which are generated by endo-reduplication from Xs that are retained—is enriched in the hallmarks of heterochromatin H3K9me2/3 and the HP1-like proteins ScoHET1 and ScoHET2 (Greciano et al. 2009). As shown in Fig. 2, H4K20me3 is also enriched at the CE indicating that the H3K9me3:HP1:H4K20me3 pathway observed at imprinted gDMRs (Fig. 1b) likely operates in *Sciara coprophila*.

**Fig. 3:**
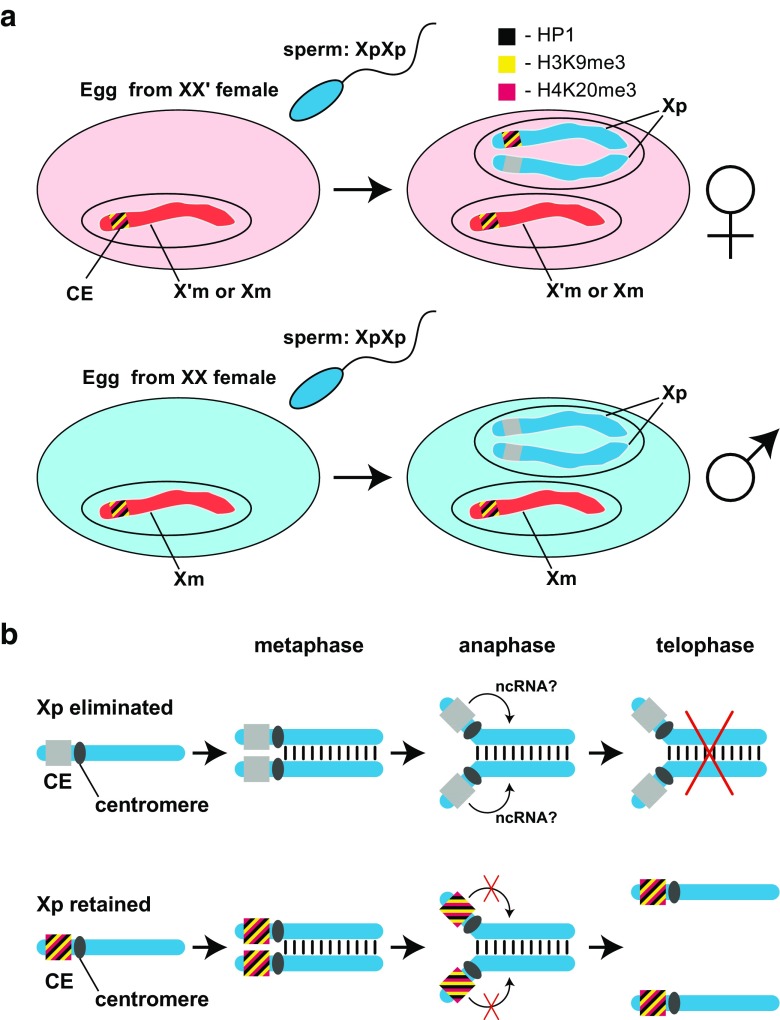
Maternal regulation of chromosomal imprinting in *Sciara coprophila.***a** Imprinting of a paternal CE in the XX′ maternal ooplasm. In *Sciara*, two types of egg are fertilized by double-X (XpXp) males (sperm nuclei are given in blue). The first type are eggs laid by XX′ females (top row on left; shaded pink) that will give rise to daughters of the genetic constitution X′mXp or XmXp because eggs conditioned by XX′ mothers eliminate one Xp in the soma. The second type are eggs laid by XX females (second row on left; shaded blue) that will give rise to XmO males because eggs conditioned by XX mothers eliminate both Xps in the soma. The Xm chromosomes are not eliminated. The controlling element (CE) on the X chromosome that is embedded within heterochromomere II adjacent to the X centromere regulates the elimination of Xps. In the top row, a model is presented where the haploid maternal nucleus laid by the XX′ mother contains either an X or X′ chromosome. The maternal CE is rendered inert by the H3K9me3:HP1:H4K20me3 pathway that assembles a heterochromatin-like complex. After the fertilization by the double-X (XpXp) sperm, the paternal pro-nucleus is formed and conditioning of the ooplasm by the XX′ mother results in assembly of heterochromatin-like complex at one of the two paternal CEs rendering it inexpressible, like the maternal CE; the complex is assembled at the CE on the Xp that will later be retained (see Fig. 3b). The remaining paternal CE is an “open” expressible state. The bottom row depicts an egg laid by the XX mother. The maternal CE is again inexpressible due to the assembly of a heterochromatin-like complex, while conditioning of the ooplasm by the XX mother leaves both paternal CEs in an “open” expressible state. The stripes of color at the CE represent H3K9me3 (yellow), H4K20me3 (purple), and HP1 protein (black). Female chromosomes are in red and male are in blue. **b** A model for the elimination of Xp chromosomes in the embryonic soma. In the top row, the paternal CE on the Xp chromosome that will be eliminated is in an “open” expressible state. This remains so after replication where the sister chromatids become aligned and connected on the metaphase plate before separation to the poles at anaphase. At anaphase, the centromeres separate, but since the CE is active, we suggest that the CE encodes an ncRNA that affects that ability of the sister chromatids to separate. As a consequence, chromatids remain physically bound together on the metaphase plate and are eliminated. The bottom row depicts the situation in embryos that develop from eggs laid by XX′ mothers where one Xp is retained. When an Xp chromosome is retained, the putative ncRNA is not expressed because of the heterochromatin-like complex assembled at the CE. Mitosis then proceeds in the orthodox manner. The chromatids align at the metaphase plate and, first, the centromeres and then the chromosome arms separate. Each chromatid then segregates into one of the daughter nuclei. The model is taken and modified from Singh (2016)

How the CE controls the somatic elimination of Xps *in cis* has been revealed by careful study of the fate of wild-type Xps and X-autosome translocations during the embryonic cleavages (de Saint Phalle and Sullivan 1996). This has shown that the CE does not affect the X centromere but rather the CE acts at-a-distance to inhibit the separation of the Xp arms at anaphase leaving the Xp chromosomes languishing at the metaphase plate to be discarded. In a synthesis of the available evidence, we suggest a testable model based on the mechanism by which long non-coding RNAs (lncRNAs) regulate mammalian autosomally imprinted genes *in cis* and over large distances (Barlow and Bartolomei 2014). Accordingly, the CE encodes a non-coding RNA (ncRNA) that acts *in cis* and at-a-distance to inhibit the separation of the Xp arms (Fig. 3b). The model posits further that expression of the ncRNA is regulated by the H3K9me3:HP1:H4K20me3 pathway that assembles a heterochromatin*-*like complex at a paternal CE in eggs conditioned by XX′ mothers (Fig. 3a). Specifically, in eggs laid by XX′ mothers, a heterochromatin*-*like complex is assembled at one paternal CE rendering it inexpressible leaving the other paternal CE expressible; assembly takes place in the paternal pro-nucleus. Thus, in the male pro-nucleus of XX′ eggs, a complex is assembled at the CE on the Xp that is to be retained (Fig. 3a). In eggs laid by XX mothers, both paternal CEs are expressible. The maternal CE is likely to have been assembled into a heterochromatin*-*like complex during oogenesis (Fig. 3a). At the 7th–8th cleavage, the expressible paternal CE(s) is activated and causes elimination of the X-chromosome(s) in *cis* (Fig. 3b). The mechanism for elimination of the Xp from the germ-line is likely to be very different to that which regulates the elimination of the Xps from the embryonic soma. First, only a single Xp is eliminated in the germ-lines of both sexes, yet two Xps are eliminated from the embryonic soma in embryos developing from eggs laid by XX androgenic mothers. This would suggest a different “counting” mechanism in the germ-line that may be related to the de-acetylated state of the Xp that is eliminated in the germ-line (Goday and Ruiz 2002). Second, resting germ cells have an intact nuclear membrane and elimination involves “passage” of the Xp through the nuclear membrane into the cytoplasm (Berry 1941; Rieffel and Crouse 1966).

The relative contribution of the germ-line and post-fertilization mechanisms to the imprinting phenomena in *Sciara* indicates a greater contribution of the egg cytoplasm to the imprinting process. However, given the advances in molecular techniques, it may be worth investigating that brief window in anaphase II of secondary spermatocytes for epigenetic modifications that are carried on the maternal (then paternal) chromosomes into the egg. That is, there might be two imprinting systems working in parallel, one for the elimination of the paternal chromosome set in primary spermatocytes and another for elimination of Xps from the soma and germ-line.

It was in coccids, also known as mealy bugs, that the role of heterochromatinization and the H3K9me3:HP1:H4K20me3 pathway in chromosomal imprinting was first described. It is to this organism we now turn, especially since it lies at one end of the spectrum where the situation seems unambiguous: the site and timing of imprinting in mealy bug is in the egg.

## Maternal regulation of chromosomal imprinting in coccids

**Fig. 4:**
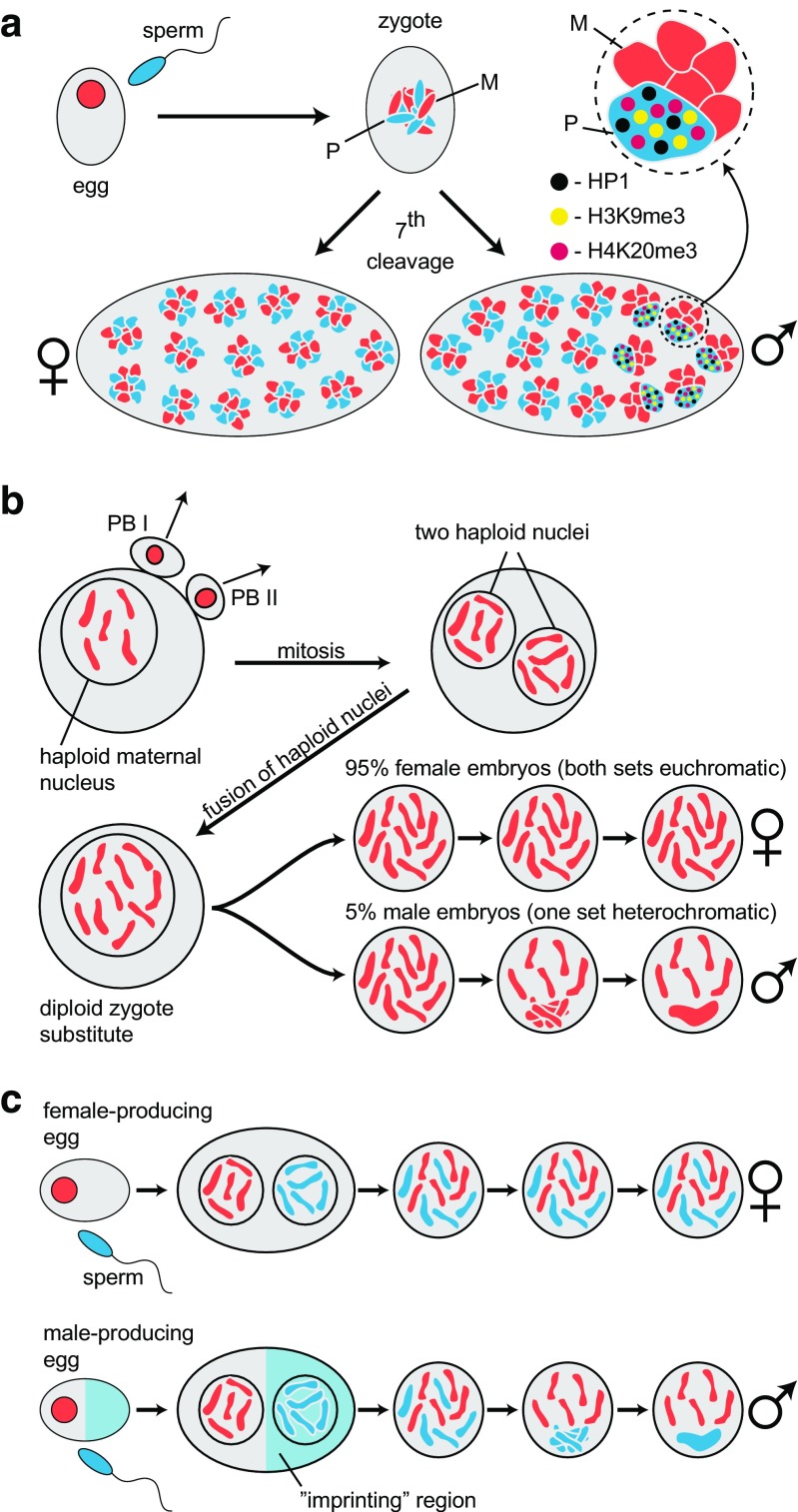
Maternal regulation of chromosomal imprinting in coccids. **a** Heterochromatinization of the paternal chromosome set in coccids. The sperm (nucleus in blue) fertilizes the egg (nucleus in red). The zygote contains 10 chromosomes (2n = 10), which is the typical number found in coccid species. At around the 7th cleavage division in male embryos, a wave of heterochromatinization begins at one of the poles (shown in male embryo beginning at the right-hand pole). Heterochromatinization leads to the formation of a chromocenter (blue) that consists of the aggregation of the paternal chromosome set, which is shown magnified in the inset above the male embryo. The chromocenter is enriched in two heterochromatin-specific histone modifications H3K9me3 (yellow) and H4K20me3 (purple) as well as the non-histone chromosomal protein HP1 protein (black). In the rest of the male embryo individual, paternal (blue) and maternal (red) chromosomes can be observed and there is no chromocenter. This is the case throughout the female embryo given on the left. The “dots” of H3K9me3 (yellow), H4K20me3 (purple), and HP1 protein (black) simply represent enrichment—their distributions overlap on the heterochromatic set in embryos (Bongiorni et al. 2007; Cowell et al. 2002; Kourmouli et al. 2004). **b** The development of parthenogenetic males and females embryos in *Pulvinaria hydrangeae*. Meiosis gives rise to a haploid maternal nucleus containing 5 chromosomes; chromosomes at all stages are given in red as they are entirely maternal in origin. Polar bodies (PBs) I and II, once extruded, take no part in the events that follow. The maternal nucleus undergoes a haploid mitosis and the products fuse to give a diploid zygote substitute, 95% of which give rise to females where both sets are euchromatic. Five percent of the embryos produce males where one set of chromosomes becomes heterochromatic. Since the males are derived without any paternal contribution, the imprinting leading to subsequent heterochromatinization is therefore under maternal control and determined by conditioning of the maternal ooplasm. **c** A model for the maternal regulation of imprinting in coccids. In the top row, the maternal nucleus (in red) lies in an oocyte cytoplasm conditioned to produce females after fertilization (sperm nucleus is given in blue). Both chromosome sets remain euchromatic at all stages of development shown. In the bottom row, oocytes conditioned to produce males the egg are spatially differentiated with a region (shaded in blue) that contains factors or determinants laid down by the mother that can “imprint” chromosomes in the paternal pro-nucleus for later heterochromatinization. Heterochromatinzation is depicted as the step-wise aggregation of the paternal chromosomes (blue) into a chromocenter. The maternal chromosomes are colored red

The chromosomal system in mealy bugs is an epigenetic *tour de force* that has fascinated cytogeneticists for many decades (reviewed in Brown and Nur (1964); Hughes-Schrader (1948)). In the newly fertilized zygote, both parental chromosome sets are euchromatic and they remain so during the first five to six cleavage divisions, which are under maternal control. It is around the seventh cleavage when transcription is first detected from the zygotic genome (Sabour 1972) and many nuclei have migrated to the periphery of the egg that heterochromatinization of the paternal chromosome set is observed in male embryos as a “wave” of heterochromatinization from one end of the embryo to other (Bongiorni et al. 2001; Bongiorni et al. 2007) (Fig. 4a). Early work revealed that heterochromatinization resulted in gene inactivity—the paternal chromosome set in male embryos is inert, while in the same nucleus, the maternal homologs are active (Brown and Nelson-Rees 1961).

The heterochromatic set becomes enriched in epigenetic markers H3K9me3 and H4K20me3, as well as the HP1-like protein, Pchet2 (Fig. 4a; Bongiorni et al. 2007; Cowell et al. 2002; Epstein et al. 1992; Kourmouli et al. 2004). A role for the H3K9me3:HP1:H4K20me3 pathway in heterochromatinzation has been tested directly. “Knock-down” of Pchet2 using dsRNA leads to a dramatic de-condensation of the heterochromatic set and dissolution of both H3K20me3 and pchet2, with the remaining H3K9me3 decorating residual blocks of heterochromatin (Bongiorni et al. 2007). These data indicate that heterochromatin is essential for regulation of the (inert) paternal chromosome set.

The search for germ-line-specific “imprints” has revealed epigenetic modifications (Bongiorni et al. 2009; Mohan and Chandra 2005) and chromatin structures (Khosla et al. 1996) peculiar to sperm chromatin although no functional work has been done to test whether these sperm-specific features can direct heterochromatinization in male embryos. Studies have also shown female-specific DNA methylation that has been suggested to “protect” the maternal genome from heterochromatinization (Bongiorni et al. 1999). The presence of germ-line-specific modifications is intriguing. However, the question of whether a germ-line specific imprint can regulate heterochromatinization had already been addressed directly using a parthenogenetic soft scale, closely related to mealy bugs, called *Pulvinaria hydrangeae* (Nur 1963). Here, embryos of both types, with and without heterochromatinization, are produced parthogenetically (Nur 1963, 1972, 1971). There is no contribution from the father. As shown in Fig. 4b, female meiosis is orthodox and gives rise to typical products. The two polar bodies that are eliminated contain euchromatic chromosomes and appear to escape “imprinting”, which is supported by observations in mealy bugs where virgin females mated to heavily irradiated males produce gynogenetic females that are derived from one or both polar bodies and possess chromosomes that are always euchromatic (Chandra 1963a, b). Notably, in *Pulvinaria hydrangeae*, the egg nucleus undergoes one round of mitosis and the haploid daughter nuclei then fuse to give rise to a diploid zygote substitute (Fig. 4b). The majority of embryos that form from this union are female possessing euchromatic chromosomes but towards the end of the egg laying cycle, males are produced that have the typical heterochromatic set (Fig. 4b). The parthenogenetic males are not functional because females were never found with sperm in their ovarian ducts (Nur 1963). Clearly, genetic factors cannot be involved because the mothers are themselves derived from the same process and thus completely homozygous. The conclusion drawn was that what was observed in the parthenogenetic species was derived from sexually reproducing species because it was unlikely that a system evolved specifically to produce useless males (Chandra and Brown 1975). The interpretation was that in sexually reproducing species, it is the ooplasm that regulates heterochromatinisation post-fertilization, most likely by “imprinting” the paternal chromosome set in the pro-nucleus prior to fusion at syngamy. The oocyte-derived imprint then causes the later heterochromatinization of the paternal chromosome set.

Based on the observations that the egg nucleus is immune from the imprinting activity in the ooplasm because the maternal chromosomes are always euchromatic and, as explained, the polar bodies are also invariably euchromatic, it was suggested that the imprinting region in the eggs is restricted (Chandra and Brown 1975). That is, females produce two types of egg (Fig. 4c). In those that will go on to give males the ooplasm of the egg is differentiated and possesses region(s) that can imprint a chromosomal set for later heterochromatinization. Once the chromosomes within a pro-nucleus or the product of haploid mitosis are exposed to the “imprinting” environment, they are imprinted and subject to heterochromatinization.

## Heterochromatin and maternal regulation of chromosomal imprinting

The maternal cytoplasm has a crucial role to play in the parent-of-origin-specific behavior of chromosomes in the imprinting systems described. The three organisms appear on a continuum. In coccids, evidence that the egg represents the site of imprinting of paternal chromosomes seems indubitable (Fig. 4b). The situation in *Sciara* is more complicated. While it remains that a paternal imprint may be carried into the egg via the sperm, the maternal ooplasm can veto any such imprint. It is sufficient for the site of imprinting to be the egg in *Sciara*, where imprinting takes place in the pro-nuclei of the newly fertilized zygote (Fig. 3a). At least for elimination of the Xp chromosomes in the soma this seems to be true. A key question is the molecular nature of the initial imprint—what is it in the maternal ooplasm that marks the Xp chromosomes? This problem is tractable now with the advent of whole genome proteomic and RNA-Seq techniques, which can be used to compare the contents of XX′ vs XX eggs. The isolation of the molecules (protein or RNA) that imprint the Xp chromosomes in *Sciara* will begin the search for conserved molecules that might undertake the same function in coccids.

At the other end of the continuum, imprinted gDMRs in the mouse confirm the role of the germ-line in chromosomal imprinting. However, what has become clear in recent years is that differential methylation is found at many loci in the genomes of the respective germ cells and imprinted gDMRs are nothing out of the ordinary; they do not represent a special class of loci that are differentially methylated in the germ lines (Kobayashi et al. 2012; Smallwood et al. 2011). What is central to imprinting in the mouse is the preservation of the imprinted gDMRs in the face of DNA demethylation in pre-implantation embryos (Messerschmidt et al. 2014). A characteristic of the sequence-specific preservation of imprinted gDMRs is that it involves the assembly of localized heterochromatin-like complexes that utilize the H3K9me3:HP1:H4K20me3 pathway (Fig. 1b). This pathway operates in coccids during heterochromatinisation of paternal chromosomes (Fig. 4a) and is also likely to assemble at the CE in *Sciara coprophila*, which controls the elimination of the Xp chromosomes in the soma (Figs. 2 and 3a, b). The H3K9me3:HP1:H4K20me3 pathway may represent the most evolutionarily conserved mechanism associated with chromosomal imprinting (Bongiorni et al. 2007; Cowell et al. 2002; Kourmouli et al. 2004; Singh 2016).

Conservation of the H3K9me3:HP1:H4K20me3 pathway is likely to be related to the mechanism by which the “imprint” regulates the parent-of-origin-specific behavior of the chromosomes. In the mouse, operation of the H3K9me3:HP1:H4K20me3 pathway as part of the heterochromatin-like complex assembled at imprinted gDMRs could regulate imprinted gene expression in two different ways. For example, assembly of the heterochromatin-like complex at the imprinted gDMR that lies 2 kb upstream of H19 in the Igf2-H19 cluster enables a downstream enhancer to act upon the Igf2 gene on the paternal chromosome and lead to its activation, while repressing the non-coding H19 RNA (Bartolomei and Ferguson-Smith 2011). This “insulator” model for imprinting of the Igf2-H19 cluster represents a special case. More often, gDMRs of imprinted gene clusters regulate the expression of lncRNA molecules that in turn regulate protein-coding genes that exhibit imprinted gene expression (Barlow and Bartolomei 2014). The lncRNA molecules act *in cis* and across clusters that can range in size from around 100 kb to 3.7 Mb (Barlow and Bartolomei 2014). As explained, the CE in *Sciara* may operate in the same way, where the assembly of a heterochromatin-like complex at one paternal CE in XX′ eggs regulates a ncRNA (Fig. 3b). The putative ncRNA encoded by the CE would act like the mammalian lncRNAs—*in cis* and over a long distance.

Studies on chromosomal imprinting are now entering an interesting phase where modern whole genome techniques are being applied to insect systems. It should not be long before molecular mechanisms are described that operate post-fertilization, and perhaps in the germ-line, to regulate parent-of-origin-specific behavior of chromosomes in these classical systems. We should also expect further conserved mechanisms to be revealed that will provide insight into chromosomal imprinting in other animals, including mouse and human.

## Abbreviations

5meC: 5-methylcytosine
CE: controlling element
CGIs: CpG islands
CpG: CpG dinucleotide
Dnmt1: maintenance DNA methyltransferase 1
dsRNA: double-stranded RNA
ES: embryonic stem
gDMRs: germ-line differentially methylated regions
H3K9me2: *di-*methylated lysine 9 on histone H3
H3K9me3: *tri-*methylated lysine 9 on histone H3
H4K20me3: *tri-*methylated lysine 20 on histone H4
HMTases: histone methyltransferases
HP1: Heterochromatin protein 1
ICR: imprinting control region
KAP1: KRAB-associated protein 1
Kb: kilobases
KRAB-Zfp: Krüppel-associated box (KRAB) domain zinc-finger protein
lncRNA: long non-coding RNA
Mb: megabases
mRNA: messenger RNA
ncRNA: non-coding RNA
PB: polar body
Pchet2: *Plannococcus citri* heterochromatin protein 2
ScoHET1: *Sciara coprophila* heterochromatin protein 1
ScoHET2: *Sciara coprophila* heterochromatin protein 2
Setdb1: SET domain bifurcated 1 K9H3 HMTase
Xp: paternal X chromosome
Xm: maternal X chromosome
Zfp57: KRAB-zinc-finger protein 57
